# Alkaline arginine promotes the gentamicin-mediated killing of drug-resistant *Salmonella* by increasing NADH concentration and proton motive force

**DOI:** 10.3389/fmicb.2023.1237825

**Published:** 2023-09-19

**Authors:** Chunyang Zhu, Yanhong Zhou, Jian Kang, Heng Yang, Jinglin Lin, Binghu Fang

**Affiliations:** ^1^Guangdong Provincial Key Laboratory of Veterinary Pharmaceutics Development and Safety Evaluation, South China Agricultural University, Guangzhou, China; ^2^National Risk Assessment Laboratory for Antimicrobial Resistance of Animal Original Bacteria, South China Agricultural University, Guangzhou, China; ^3^School of Animal Science and Technology, Guangdong Polytechnic of Science and Trade, Guangzhou, China

**Keywords:** Salmonella, arginine, gentamicin, resistance, proton motive force

## Abstract

**Introduction:**

Antimicrobial resistance, especially the development of multidrug-resistant strains, is an urgent public health threat. Antibiotic adjuvants have been shown to improve the treatment of resistant bacterial infections.

**Methods:**

We verified that exogenous L-arginine promoted the killing effect of gentamicin against *Salmonella in vitro* and *in vivo*, and measured intracellular ATP, NADH, and PMF of bacteria. Gene expression was determined using real-time quantitative PCR.

**Results:**

This study found that alkaline arginine significantly increased gentamicin, tobramycin, kanamycin, and apramycin-mediated killing of drug-resistant *Salmonella*, including multidrug-resistant strains. Mechanistic studies showed that exogenous arginine was shown to increase the proton motive force, increasing the uptake of gentamicin and ultimately inducing bacterial cell death. Furthermore, in mouse infection model, arginine effectively improved gentamicin activity against *Salmonella typhimurium*.

**Discussion:**

These findings confirm that arginine is a highly effective and harmless aminoglycoside adjuvant and provide important evidence for its use in combination with antimicrobial agents to treat drug-resistant bacterial infections.

## 1. Introduction

Infections with drug-resistant bacteria, including multidrug-resistant (MDR) strains, are becoming a greater threat to human health and the farming industry. The emergence of a few superbugs has cost most extant antimicrobials to lose their efficacy (Ciorba et al., 2015). *Salmonella* spp. is a common and widely distributed zoonotic pathogen that can cause severe disease in both humans and animals. The more recent spread of MDR *Salmonella* spp. has made it more difficult to contain these infections (Hogberg et al., 2010). Thus, there is an urgent need to develop measures to contain the occurrence and spread of drug-resistant bacteria.

Antibiotic adjuvants, substances that do not have their own antibacterial effects but can increase the potency of antibiotics, are shown to be effective against drug-resistant microbes (Liu et al., 2019a). Clavulanate and sulbactam are prominent adjuvants that increase the effect of penicillin by inhibiting β-lactamase. Given that the metabolic status of bacteria impacts antimicrobial efficacy, metabolic regulators (Stokes et al., 2019), a class of antibiotic adjuvants, used in combination with antimicrobial agents, is considered a potential strategy for treating MDR bacteria (Liu et al., 2019b).

Metabolic regulators were first used to treat *Escherichia coli* and *Staphylococcus aureus* persisters (Allison et al., 2011). Specifically, glucose, fructose, mannitol, or pyruvate were used to fuel bacterial glycolysis, promote NADH generation, activate the electron transport chain (ETC), and increase the cell membrane proton motive force (PMF), facilitating the uptake of gentamicin and ultimately leading to bacterial cell death. Peng et al. (2015) demonstrated that alanine, glucose, or fructose promote kanamycin activity using a similar mechanism against drug-resistant *Edwardsiella tarda* (Su et al., 2015). Several metabolic regulators are also shown that enhance the activity of beta-lactam, fluoroquinolones, and aminoglycosides against *E. coli*, *Pseudomonas aeruginosa*, *Klebsiella pneumoniae*, and *Vibrio alginolyticus* (Meylan et al., 2017; Liu et al., 2021; Zhao et al., 2021). To date, however, few studies have identified metabolic regulators that synergize with antimicrobial agents used to treat *Salmonella* spp., including persisters and drug-resistant cells.

Aminoglycosides are highly potent and broad-spectrum antimicrobials that are used to treat a wide range of pathogens (Poulikakos and Falagas, 2013). However, the PMF of bacterial cell membranes is needed to aid the internalization of these drugs (Taber et al., 1987). Our previous study found that some amino acids and sugars can regulate the metabolic state of bacteria and promote PMF, eventually increasing the uptake of aminoglycosides (Yong et al., 2021; Zhou et al., 2022). Alkalinized media can also provide bacterial cells with a transmembrane pH difference (ΔpH) and increase PMF (Sabath and Toftegaard, 1974; Letoffe et al., 2014). For example, lysine, a basic amino acid that can confer the transmembrane difference in H + concentration to bacteria, is shown to increase aminoglycoside-mediated killing of MDR *Acinetobacter baumannii*, *Escherichia coli*, and *Klebsiella pneumoniae* (Deng et al., 2020). Similarly, alkaline arginine promotes the activity of gentamicin against *Staphylococcus aureus*, *E. coli*, and *Pseudomonas aeruginosa* persisters both *in vitro* and *in vivo*. The existence of pH-independent factors that promote arginine-mediated antimicrobial activity has also been proposed (Lebeaux et al., 2014). Further studies are needed to identify the mechanism by which arginine synergizes with antibiotics.

Our previous study confirmed that arginine biosynthesis and metabolism are disrupted in gentamicin-resistant *Salmonella*, and arginine levels are reduced in resistant cells. D-ribose, which facilitates gentamicin-mediated killing of drug-resistant *Salmonella*, was similarly suppressed in resistant bacteria (Zhou et al., 2022). Thus, we speculated that arginine could promote gentamicin activity against drug-resistant *Salmonella*.

The current study confirmed that the basic amino acid, arginine, can promote the activity of aminoglycosides against drug-resistant *Salmonella* spp. including clinically isolated MDR strains. Arginine was able to enhance aminoglycoside function by affecting the electrical potential across the membrane (Δψ) and the transmembrane difference in H + concentration (ΔpH). The findings confirm that arginine is a highly effective and harmless aminoglycoside adjuvant and further previous findings of arginine-mediated antimicrobial potentiation. Overall, this study provides important evidence for the use of arginine in combination with antimicrobial agents to combat drug-resistant bacteria.

## 2. Materials and methods

### 2.1. Chemicals

All antibiotics were purchased from Shanghai Macklin Biochemical Technology Co., Ltd (Shanghai, China). Mueller Hinton (MH) Agar, MH Broth, Tryptic Soy Agar (TSA), Luria-Bertani (LB) broth, and MacConkey Agar were purchased from Guangdong Huankai Microbial Sci & Tech. Co., Ltd. (Guangdong, China). Arginine purchased from Beijing Solarbio Science & Technology Co., Ltd. (Beijing, China). Mouse serum were purchased from Guangzhou Ruite Biotechnology Co., Ltd.

### 2.2. Bacterial strains and growth conditions

*Escherichia coli* (ATCC25922) Standard strain was purchased from American Type Culture Collection (Manassas, VA, United States). *Salmonella* typhimurium (CICC 21484) Standard strain was purchased from China Center of Industrial Culture Collection (Beijing, China). The standard *S.* typhimurium (CICC 21484) strain was sequentially propagated with or without gentamicin and drug-resistant bacteria were selected (STM-R). The clinical strains, SR-1 (*S.* Derby), SR-2 (*S.* 1, 4, [5], 12: i:-), SR-7 (*S.* typhimurium), and SR-8 (London) were isolated from swine farms in South China (Guangdong Province, China). These strains confer resistance to most clinical antimicrobials, including β-lactams, aminoglycosides, tetracyclines, and sulfonamides (Table 1).

**TABLE 1:** MIC value of different antimicrobials against *Salmonella* (μg/mL).

	STM-S	STM-R	SR-1	SR-2	SR-7	SR-8
Gentamicin	0.25	32	32	64	32	64
Tobramycin	0.5	8	32	64	32	32
Kanamycin	1	4	512	256	512	32
Apramycin	2	8	128	128	128	128
Ampicillin	2	2	128	512	512	64
Colistin	0.5	0.5	1	0.5	1	1
Sulfisoxazole	2	2	512	512	512	512
Tetracycline	0.5	0.5	32	128	32	64
Florfenicol	1	1	256	256	128	256
Sulfamethoxazole/trimethoprim	0.5/9.5	0.5/9.5	4/76	4/76	4/76	4/76
Cefotaxifur	0.25	0.25	1	0.5	0.5	1
Ciprofloxacin	0.05	0.05	8	0.5	8	0.5
Enrofloxacin	0.03	0.03	4	1	4	0.5
Amoxicillin/clavulanic acid	2/1	2/1	32/16	16/8	16/8	32/16
Meropenem	0.05	0.05	0.03	0.03	0.03	0.03
Ceftazidime	0.5	0.5	1	0.5	0.5	0.25

### 2.3. Antibiotic bactericidal assays

The antibacterial assay was carried out as described previously (Allison et al., 2011). In brief, bacterial cells were cultured in LB broth for 8 h. After centrifugation at 13,000 × *g* for 5 min at 4°C, the precipitate was washed twice with sterile PBS buffer and resuspended to 1 × 10^6^ colony-forming units (CFU)/mL in M9 minimal media. Gentamicin-resistant *Salmonella* was inoculated into 3 mL of M9 broth containing gentamicin, gentamicin plus arginine, respectively. For all CCCP experiments, the cells were preincubated with 20 μM CCCP for 5 min before the addition of metabolites or antibiotics. The remaining inhibitors, malonate, and rotenone were added at the same time as the metabolites or antibiotics. The bacteria were counted at 0, 2, 4, 6, and 8 h, and bacterial survival curves were drawn. Percentage survival was determined by the ratio of CFU obtained from the test and control samples. To investigate the effect of arginine on the bactericidal activity of Gentamicin against *Salmonella* in mouse serum culture medium. The experimental strains (STM-R, SR-1, SR-2, SR-7, and SR-8) were treated with Gentamicin (1 MIC), and with or without arginine (10 mM) for 8 h in mouse serum medium.

### 2.4. Synergistic antibacterial effect in a mouse infection model

The mouse infection model was established according to previously described methods (Yong et al., 2021). The 6-week-old female KM mice (body weight b.w., 18–22 g) were intraperitoneally injected with *Salmonella* typhimurium STM-R and SR-7 (10^7^ CFU/mL for both), mice were divided into 4 groups, 6 mice in each group. Investigate the therapeutic effect of arginine combined with gentamicin on a mouse infection model. Investigate treatment with 0.9% saline (control, 0.1 mL), arginine (100 mg/kg b.w.), gentamicin (10 mg/kg b.w.), and arginine plus gentamicin (100 mg/kg plus10 mg/kg b.w.), administered the same dose 12 h after the first administration. Twelve hours after the second administration, six mice were randomly selected and sacrificed by cervical dislocation. The blood, liver, kidney and spleen samples of mice were collected for bacterial count, and the bacterial load of each organ was counted to investigate the therapeutic effect of arginine combined with gentamicin on mice. Ethics Statement: This study was approved by the Animal Ethics Committee of South China Agricultural University (application NO:2023b020) and was carried out in accordance with the ARRIVE guidelines.

### 2.5. Measurement of NADH and ATP

The intracellular NADH concentration of *Salmonella* was detected as previously described (Peng et al., 2015). NADH concentration was detected using an NAD + /NADH assay kit (BioAssay Systems, Hayward, CA, United States). The bacteria were cultured in M9 medium with or without a final concentration of 10 mM arginine for 8 h. After that, 1 mL of the bacterial solution was collected and centrifuged at 13,000 × *g* for 5 min at 4°C to collect the precipitate. The supernatant was discarded, and the pellet was washed three times with PBS. NADH extraction buffer (100 μL) was added, the extract was heated at 60°C for 5 min, and 20 μL assay buffer and 100 μL NAD + extraction buffer were added to neutralize the extract. After centrifugation, NADH was measured in the supernatant according to the manufacturer’s instructions. ATP concentration was measured using an ATP assay kit (Beyotime, China) as described previously (Liu et al., 2021). After co-culturing the bacteria (1 × 10^6^ CFU/mL) and 10 mM arginine for 4 h, the bacterial solution was centrifuged at 4°C and 12,000 × *g* for 5 min. The supernatant was discarded, and the pellet is carefully resuspended in PBS and washed twice. ATP levels were determined using a commercially available kit following the manufacturer’s instructions.

### 2.6. Measurement of membrane potential

Arginine was added into the M9 medium of Gentamicin-resistant *Salmonella*. The mixture was then incubated at 37°C for 8 h. After incubation, 1 mL of bacterial solution was removed and centrifuge at 20,000 × *g* for 5 min at −10°C. The supernatant was discarded. The precipitate was washed twice with PBS and then diluted to approximately 1 × 10^6^ CFU/mL with PBS. The membrane potential was examined using a BacLight Bacterial Membrane Potential Kit (Life Technologies, Carlsbad, CA, United States) as previously described (Zhou et al., 2022).

### 2.7. Measurement of intracellular gentamicin

Intracellular gentamicin levels were determined as previously described (Zhou et al., 2022). In brief, bacterial cells were cultured in LB broth until the late exponential phase. After centrifugation, the precipitates were collected and resuspended in 10 mL of M9 minimal media to 1 × 10^6^ CFU/mL with or without antibiotics and arginine. The reaction samples were incubated at 37°C for 6 h, and the precipitates were harvested by centrifugation, washed twice with PBS, and resuspended in 1 mL of PBS. The solution was sonicated for 10 min and the insoluble substances were removed by centrifugation. Gentamicin was quantified in the supernatant using a gentamicin ELISA kit (Shanghai Enzymelinked Biotechnology Co., Ltd., Shanghai, China).

### 2.8. Quantitative RT-PCR analysis

Bacteria were cultured in 50 mL M9 medium with or without 10 mM arginine for 8 h. The bacteria cells were collected following centrifugation at 8,000 rpm for 10 min (4°C, discard the supernatant). The pellet washed three times with sterile PBS buffer. The bacterial samples were snap frozen by liquid nitrogen and stored at −80°C until analysis. RT-qPCR assay was in agreement with the previous method (Wang et al., 2023). The specific primers are listed in the (Supplementary Table 1).

### 2.9. Statistical analysis

Results are presented as means ± SD. The statistical analysis was performed using SPSS Statistics 26.0 software (IBM Corp., Armonk, NY, United States). Statistical significance was set as *p < 0.05, **p < 0.01 (use the *t*-test or one-way ANOVA). Three biological repeats were carried out.

## 3. Results

### 3.1. Exogenous arginine promotes aminoglycoside activity against drug-resistant *Salmonella*

**FIGURE 1:**
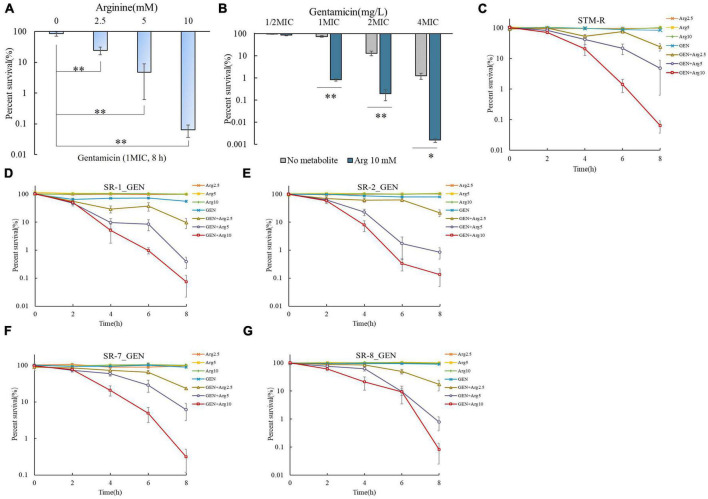
Arginine induces gentamicin-mediated killing in drug-resistance *Salmonella*. **(A)** Percent survival of STM-R in the presence of the indicated concentration of arginine and 1 MIC gentamicin. **(B)** Percent survival of STM-R in the presence of the indicated concentration of gentamicin and with or without 10 mM arginine. Percent survival of lab-evolved STM-R **(C)** and clinical isolates SR-1 **(D)**, SR-2 **(E)**, SR-7 **(F)**, and SR-8 **(G)** in the presence of gentamicin(1MIC) by arginine dose. Results are displayed as the mean ± SD and three biological repeats are carried out. Significant differences are identified (*p < 0.05 and **p < 0.01 as determined by *t*-test).

Exogenous arginine promotes the gentamicin-mediated killing of *E. coli* and *Staphylococcus aureus* persister cells both *in vitro* and *in vivo*. We speculated that arginine can also promote gentamicin-mediated killing of drug-resistant *Salmonella*. Four clinical *Salmonella* isolates with different serotypes, including *Salmonella* typhimurium, London, Derby, and 1, 4, [5], 12: i: -, which are resistant to almost all common antibiotics, were used to test this hypothesis. Arginine was shown to significantly increase the killing of gentamicin-resistant *Salmonella* in a dose-dependent manner. Specifically, STM-R cell viability was reduced 3.48-, 17.85-, and 1327.29-fold when treated with 2.5, 5, and 10 mM arginine plus 1 MIC gentamicin, respectively. When the fixed arginine concentration at 10 mM, the survival rate of STM-R decreases with the increase of Gentamicin concentration. In addition, STM-R cell viability was reduced by increasing doses of gentamicin with or without 10 mM arginine (Figures 1A–C). Arginine also enhanced gentamicin activity against clinically isolated MDR *S.* Derby (SR-1), *S.* 1, 4, [5], 12: i:–(SR-2), *S.* typhimurium (SR-7) and *S.* London (SR-8) (Figures 1D–G), and increased tobramycin-, kanamycin-, and apramycin-mediated killing of MDR *Salmonella* (Supplementary Figures 1–3). Furthermore, the efficacy of the treatment was also observed in mouse serum. Mouse serum showed little or no clearance of the different Salmonella strains (compared with M9 medium). When no substance was added, the number of bacteria in the two media (M9 and serum) was increased to the power of 108 CFU/mL after 8 h, although the serum group showed a small inhibitory effect. The statistical analysis did not constitute a significant difference (*T*-test). When treated with a combination of 10 mM arginine and 1 MIC gentamicin, the viability of *Salmonella* of different serotypes decreased significantly. The survival rates of STM-R, SR-1, SR-2, SR-7, and SR-8 decreased by 1584.42-, 1126.98-, 72.49-, 761.90-, and 1027.03-fold, respectively (Supplementary Figure 4). The arginine-potentiated aminoglycoside-mediated killing of drug-resistant *Salmonella* was both dose and incubation period-dependent. These findings show that arginine could effectively enhance aminoglycoside activity against MDR *Salmonella*.

### 3.2. Synergistic antibacterial effect in a mouse infection model

**FIGURE 2:**
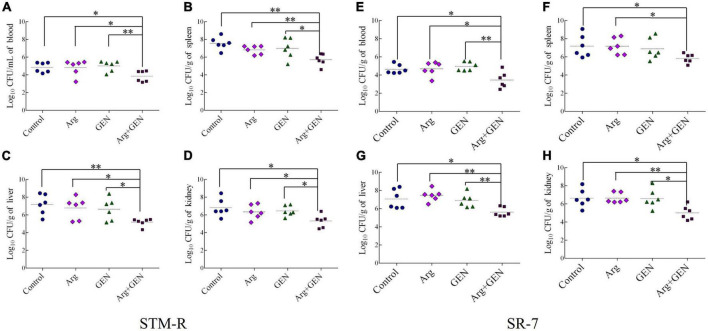
Arginine induces gentamicin-mediated killing in a mouse infection model with drug-resistance *Salmonella*. Bacterial density of blood **(A)**, spleen **(B)**, liver **(C),** and kidney **(D)** in mice infected with STM-R after treatment with different therapeutic schedule. Bacterial density of blood **(E)**, spleen **(F)**, liver **(G),** and kidney **(H)** in mice infected with SR-7 after treatment with different therapeutic schedule. Results are displayed as the mean ± SD and three biological repeats are carried out. Significant differences are identified (*p < 0.05 and **p < 0.01 as determined by *t*-test).

Mice were injected intraperitoneally with *Salmonella* typhimurium STM-R and SR-7 to establish a mouse model of drug-resistant bacterial infection. To investigate the potential synergistic effect of gentamicin and arginine in eliminating *Salmonella* typhimurium in mice. The bacterial loads were monitored, respectively, in the liver, spleen, kidney and blood. In a mouse model of infection with STM-R, the results show that the arginine plus gentamicin (test group) were significantly lower than in those treated with gentamicin alone. Compared with gentamicin alone, the bacterial load of blood, spleen, liver and kidney was reduced by 14.5, 18.2, 28.8, and 13.5-fold, respectively. At SR-7 mouse infection model, the test group had similar results (Figures 2A–H). These results indicate that arginine promotes gentamicin against drug-resistant *Salmonella in vivo*, including lab-evolved and clinically isolated multidrug-resistant *Salmonella*.

### 3.3. Arginine-induced PMF enhances gentamicin uptake

**FIGURE 3:**
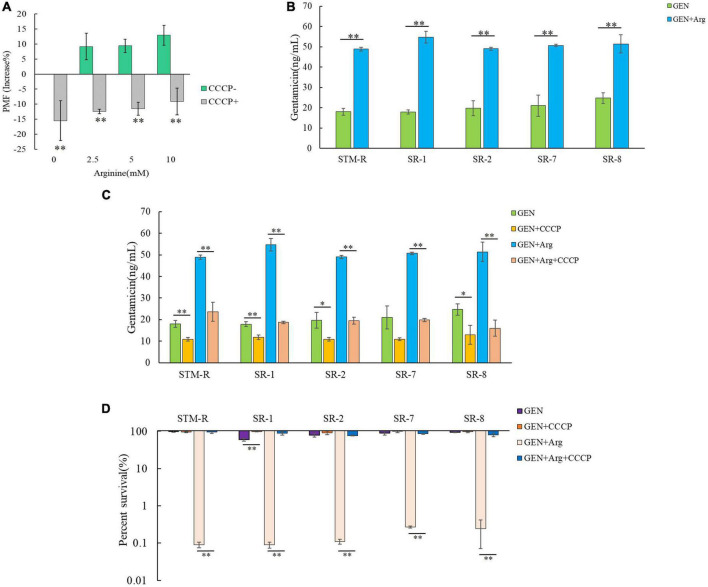
Effect of exogenous arginine on ponton motive force of *Salmonella*. **(A)** Variation of PMF in STM-R by arginine dose with or without CCCP. **(B)** Intracellular concentration of gentamicin in STM-R and clinical isolates in the presence of 10 mM arginine. **(C)** Intracellular concentration of gentamicin in STM-R and clinical isolates in the presence of 10 mM arginine with or without CCCP. GEN, gentamicin. **(D)** Percent survival of STM-R and clinical isolates in the presence or absence of CCCP and 1 MIC gentamicin plus 10 mM arginine. GEN, gentamicin. Results are displayed as the mean ± SD and three biological repeats are carried out. Significant differences are identified (*p < 0.05 and **p < 0.01 as determined by *t*-test).

Exogenous metabolites promote the uptake of aminoglycosides by increasing the bacterial cell membrane PMF and causing cell death. Thus, several assays were performed to confirm whether arginine could increase the PMF of drug-resistant *Salmonella*. Drug-resistant cells were cultured in M9 minimal media with or without arginine for 8 h and the PMF was assessed using the BacLight bacterial membrane potential lit. The PMF of STM-R increased by 9.17, 9.4, and 12.93% in response to 2.5, 5, and 10 mM arginine, respectively. In contrast, PMF decreased by 15.49, 12.43, 11.53, and 9.14% in response to 20 μM CCCP and 2.5, 5, or 10 mM arginine, respectively (Figure 3A). CCCP is a proton ionophore that inhibits PMF. Similar results were obtained when clinical MDR *Salmonella* cells were incubated with various doses of exogenous arginine (Supplementary Figure 5). These results indicated that arginine was able to enhance the PMF of drug-resistant *Salmonella*. To confirm that arginine-potentiated PMF facilitated drug uptake by bacterial cells, intracellular drug levels were measured using a gentamicin ELISA kit. The intracellular concentration of gentamicin in STM-R and MDR isolates increased significantly in the presence of 10 mM arginine (Figures 3B, C). The gentamicin-mediated killing was abolished by CCCP in the presence and absence of arginine (Figure 3D). These results indicated that arginine-potentiated PMF facilitated drug uptake by bacterial cells and induced cell death.

### 3.4. pH-dependent increase in PMF causes bacterial cell death

**FIGURE 4:**
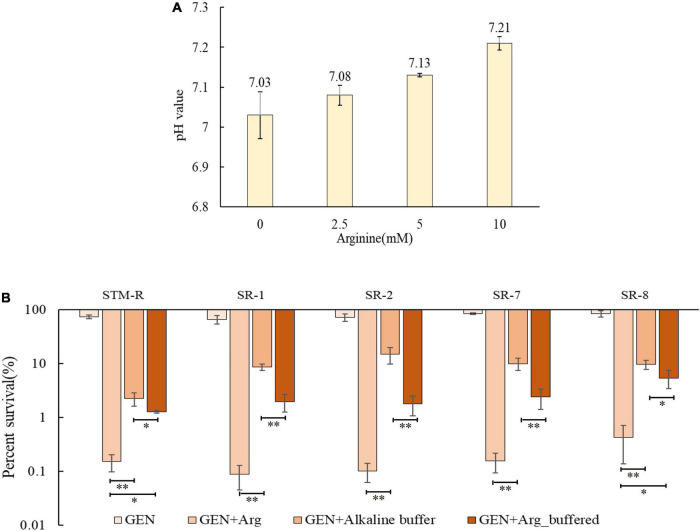
pH-induced elevated PMF promotes gentamicin-mediated killing. **(A)** pH value of M9 media with different dose arginine. **(B)** Percent survival of *Salmonella* in M9 media with buffered arginine and alkalized. Results are displayed as the mean ± SD and three biological repeats are carried out. Significant differences are identified [*p < 0.05 and **p < 0.01 as determined by one way-ANOVA, **(B)**].

The PMF is composed of both the electrical potential across the membrane (Δψ) and the transmembrane difference in H + concentration (ΔpH). The basic amino acids arginine and lysine can increase the PMF of bacterial cells depending on the ΔpH. Thus, it is probable that arginine can alkalinize media, inducing PMF and leading to cell death. To test this, drug-resistant *Salmonella* cells were incubated for 8 h in alkalinized medium with AMPSO buffer adjusted to a pH of ∼7.21 (a pH value equivalent to 10 mM arginine) (Figure 4A). Cell viability was significantly reduced in the alkalinized media containing gentamicin, suggesting that the increased pH promoted the gentamicin-mediated killing of drug-resistant *Salmonella*. However, the pH-mediated potentiation of gentamicin was weaker than the arginine-mediated potentiation even when the pH value was the same. On the other hand, buffered arginine (pH = 7.0) was able to enhance gentamicin-mediated killing (Figure 4B). These findings suggest that basic arginine may provide the ΔpH to enhance the PMF of the cell membrane and that some pH-independent factors may also be involved in this process.

### 3.5. The activated electron transport chain is responsible for increasing the PMF

**FIGURE 5:**
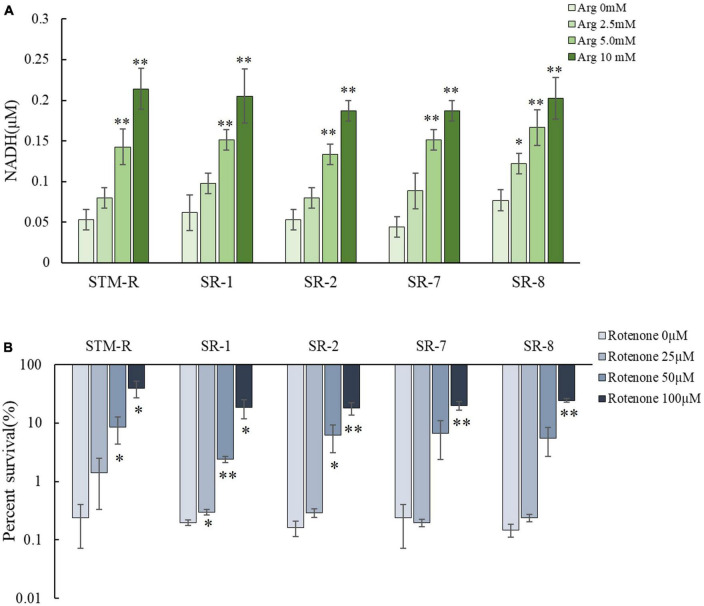
Arginine activates the ETC. **(A)** The intracellular NADH concentration of *Salmonella* by different arginine dose and 1 MIC gentamicin. **(B)** Percent survival of *Salmonella* by rotenone dose and 1 MIC gentamicin plus 10 mM arginine. Results are displayed as the mean ± SD and three biological repeats are carried out. Significant differences are identified (*p < 0.05 and **p < 0.01 as determined by *t*-test).

This study confirmed that pH-independent factors are involved in promoting the gentamicin-mediated killing of drug-resistant *Salmonella*. Several studies have shown that amino acids and sugars can elevate intracellular NADH, which activates the ETC and elevates the PMF. To test this in drug-resistant *Salmonella*, intracellular NADH levels were measured and shown to increase in response to arginine in a dose-dependent manner. While intracellular NADH levels increased markedly in response to 5 and 10 mM arginine, the increase was less apparent in response to 2.5 mM arginine (Figure 5A). This finding indicated that the arginine-mediated increase in NADH involved a threshold arginine dose. In addition, the ETC inhibitor, rotenone, partially abolished the arginine-potentiated gentamicin-mediated killing of drug-resistant *Salmonella* (Figure 5B). When the electron transport chain pumps electrons across the membrane to form a potential difference, ATP provides energy as the end product of the electron transport chain life activity. NADH as a substrate in the electron transport chain, is also affected by arginine, which may increase the intracellular ATP concentration. The intracellular ATP concentration was measured, as shown in the Supplementary Figure 6, the intracellular ATP concentration was significantly increased after arginine addition compared to the control group. These results suggested that exogenous arginine can elevate intracellular NADH, it promotes ATP production and enhances bacterial metabolic activity, which activates the ETC and ultimately elevates the PMF.

### 3.6. Arginine fuels the TCA cycle and drives aminoglycoside-mediated killing

**FIGURE 6:**
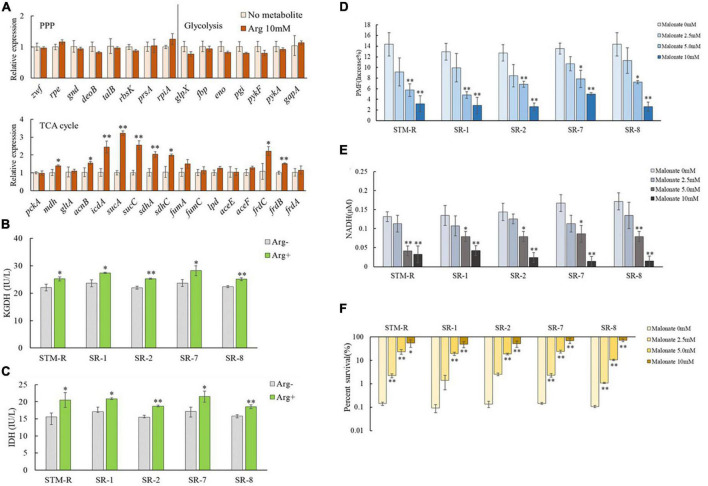
Effect of exogenous arginine on central carbon metabolism. **(A)** QRT-PCR for expression of key central carbon metabolism genes in the presence of 10 mM arginine. **(B,C)** Activity of IDH and KGDH in STM-R and clinical isolates with or without arginine. **(D–F)** The PMF, intracellular NADH and percent survival of *Salmonella* by malonate dose with 10 mM arginine. Results are displayed as the mean ± SD and three biological repeats are carried out. Significant differences are identified (*p < 0.05 and **p < 0.01 as determined by *t*-test).

Intracellular NADH is generated through partial reactions in central carbon metabolism (the TCA cycle, glycolysis/gluconeogenesis, pentose phosphate pathway) and ultimately undergoes redox reactions to activate the ETC, facilitating the PMF of the cell membrane. Thus, it was assumed that arginine activates central carbon metabolism to promote the generation of NADH. To test this, qRT-PCR was used to examine genes involved in central carbon metabolism (17 associated with the TCA cycle, 7 associated with glycolysis/gluconeogenesis, and 8 involved in the pentose phosphate pathway). Genes involved in glycolysis/gluconeogenesis and the pentose phosphate pathway remained unchanged, while nine genes involved in the TCA cycle, *mdh*, *acnB*, *icdA*, *sucA*, *sucC*, *sdhA*, *sdhC*, *frdC*, and *frdB* were upregulated in the presence of 10 mM arginine (Figure 6A). Of these, *mdh*, *icdA*, and *sucA* encoded malate dehydrogenase, isocitrate dehydrogenase, and α-ketoglutarate dehydrogenase, respectively, which are involved in NADH generation during the TCA cycle. The enzymes encoded by *sdhA*, *sdhC*, *frdB*, and *frdC* are involved in the ETC and are important for maintaining membrane potential. QRT-PCR results showed that arginine is involved in activating the TCA cycle but not the glycolysis/gluconeogenesis and pentose phosphate pathways. We also assessed the enzymatic activity of intracellular isocitrate dehydrogenase (IDH) and α-ketoglutarate dehydrogenase (KGDH) and found that the activity of both enzymes in drug-resistant *Salmonella* was elevated in the presence of 10 mM arginine (Figures 6B, C).

The TCA cycle inhibitor, malonate, which competitively inhibits succinate dehydrogenase, was used to further verify the impact of the TCA cycle on arginine-mediated potentiation. Drug-resistant *Salmonella* was incubated for 8 h in M9 media with 10 mM arginine plus malonate. As expected, arginine induced the PMF, and NADH was inhibited by malonate in a dose-dependent manner. The arginine-mediated potentiation of gentamicin was similarly inhibited by malonate. Malonate increased drug-resistant *Salmonella* cell viability in a dose-dependent manner even in the presence of arginine and gentamicin (Figures 6D–F).

**FIGURE 7:**
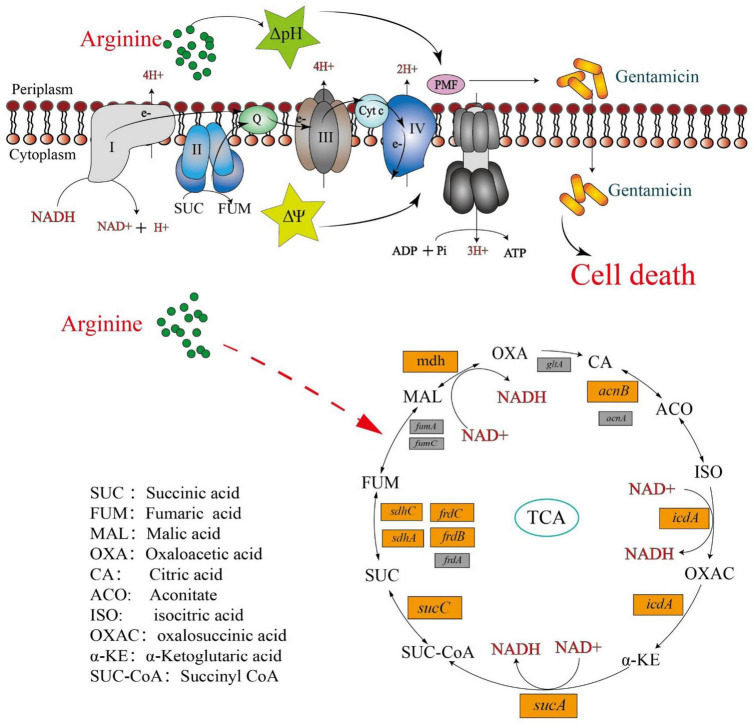
Mechanism of exogenous alkaline arginine promotes gentamicin treatment of drug-resistant *Salmonella*.

These findings suggested that in addition to providing ΔpH to increase the PMF, arginine affects the *Salmonella* TCA cycle, inducing NADH and polarizing the ETC (Figure 7). This increases the PMF and promotes drug uptake and cell death.

## 4. Discussion

There is a critical need for the development of effective strategies to cope with worsening antimicrobial resistance. The use of antibiotic adjuvants may be a better alternative to the development of new antimicrobial agents for the treatment of drug-resistant bacterial infections. This study found that basic arginine significantly increased the gentamicin-, tobramycin-, kanamycin-, and apramycin-mediated killing of drug-resistant *Salmonella*, including MDR strains. The findings provide evidence for the use of arginine as a harmless and highly effective antibiotic adjuvant. Interestingly, arginine also exhibits a positive synergistic effect in mice. However, the metabolic mechanism of arginine *in vivo* is still unclear, which is a topic worth further research.

At first, we tried to explore the mechanism of synergistic effect of arginine and gentamicin by inhibiting arginine decomposition. We conducted experiments using two inhibitors, L-NMMA and BEC. However, the results obtained from these experiments did not provide an explanation for the mechanism under study (Supplementary Figure 7). So, we selected the succinate dehydrogenase inhibitors (malonate), ETC inhibitors (rotenone) and PMF uncoupler (CCCP) for verification selected by many studies. These reagents have been proven to be reliable through several studies, with the relevant literature being referenced in the article. We used three inhibitors to elucidate our mechanism, suggesting that the synergistic effect of arginine is closely related to PMF. Bacterial cell membrane proton motive force (PMF), which is composed of ΔpH (the transmembrane pH difference) and Δψ (the electrical potential across the membrane), is required for the internalization of aminoglycosides (Taber et al., 1987; Sun et al., 2011). Thus, a rise in PMF that occurs due to changes in the ΔpH and/or Δψ can promote the antibacterial effects of aminoglycosides. This is illustrated by the increased susceptibility of bacteria to aminoglycosides in alkaline media and their decreased susceptibility to aminoglycosides in acidic media (Sabath and Toftegaard, 1974; Letoffe et al., 2014). Interestingly, while a dose-dependent effect of arginine was observed *in vitro*, a dose-dependent effect of PMF was not evident. These interesting results may prompt us that PMF is not the only mechanism by which arginine promotes killing effects. However, it is worth noting that the addition of arginine did lead to an increase in PMF, indicating that arginine does plays a significant role in elevating PMF. Indeed, Lebeaux et al. (2014) reported that basic arginine can alkalinize media and increase gentamicin-mediated killing of *E. coli* persisters and *S. aureus* biofilms both *in vitro* and *in vivo*. Alkaline lysine is also shown to assist aminoglycosides in eliminating drug-resistant *Acinetobacter baumannii*, *E. coli*, and *Klebsiella pneumoniae* (Deng et al., 2020). These studies indicate that arginine and lysine can increase aminoglycoside potentiation depending on the ΔpH. The current study similarly found that basic arginine could alkalize media, and promote gentamicin, tobramycin, kanamycin, and apramycin activity against drug-resistant *Salmonella*. These findings suggest that the use of basic substances such as arginine or lysine, to alkalize media, is an effective strategy to improve the activity of aminoglycosides.

Distinct from the ΔpH-induced PMF increase, the Δψ-dependent increase results from a series of biochemical reactions in bacterial cells (Gao et al., 2019). The Δψ, a part of the PMF, improves aminoglycoside activity. Many metabolites also increase the efficacy of aminoglycosides against persisters or drug-resistant bacteria, including glucose, fructose, alanine, and glutamic acid (Allison et al., 2011; Peng et al., 2015). Metabolite-mediated potentiation works by promoting cells to generate more NADH, activating ETC and providing the Δψ needed to elevate the PMF. While Lebeaux et al. (2014) attributed basic arginine-mediated enhancement to the ΔpH, buffered arginine was also found to partially increase gentamicin-mediated killing. The current study found that buffered arginine also promoted the activity of gentamicin against *Salmonella*, suggesting that there is a pH-independent mechanism for arginine-mediated fortification. Interestingly, the ETC inhibitor, rotenone, partially saved the *Salmonella* cells, highlighting the direct effect of membrane potential on aminoglycoside activity. These results suggest that in addition to pH, Δψ can aid arginine-mediated aminoglycoside function.

Central carbon metabolism, which includes glycolysis, the pentose phosphate pathway (PPP), and the TCA cycle, is required for the generation of intracellular NADH (Baughn and Rhee, 2014; Dolan et al., 2020). Glucose, fructose, and pyruvate entering glycolysis can promote intracellular NADH production in *E. coli*, activate ETC, and induce the PMF. However, the entry of D-ribose into the PPP does not have a similar effect (Allison et al., 2011). Interestingly, our previous study found that D-ribose provides NADH to drug-resistant *Salmonella* through feeding the PPP, and then activates and finally promotes gentamicin activity (Zhou et al., 2022). Peng et al. (2015) demonstrated that metabolites such as alanine, glutamate, fructose, and glucose were able to stimulate the TCA cycle in drug-resistant *Edwardsiella tarda*, promote NADH production, and induce higher PMF to facilitate the uptake of kanamycin (Su et al., 2015). These studies suggested that central carbon metabolism is an important target for metabolite-mediated antimicrobial potentiation. To determine whether arginine influence central carbon metabolism in drug-resistant *Salmonella*, qPCR was performed for genes associated with this pathway in the presence or absence of arginine. Arginine was shown to fuel the TCA cycle but not glycolysis and PPP. Thus, we hypothesized that alkaline arginine provides both ΔpH and activates ETC through the TCA cycle for resistant cells, increasing the PMF of resistant bacteria using both the ΔpH and Δψ, promoting the uptake of gentamicin, and ultimately causing bacterial death.

In summary, arginine is a crucial amino acid that plays an indispensable role in promoting growth and development. The research has indicated that arginine exhibits superior immunomodulatory and anti-inflammatory properties (Kuo et al., 2018; Chen et al., 2022). We developed a strategy for eradicating drug-resistant *Salmonella* and provided evidence to support its use. Arginine was shown to serve as an effective aminoglycoside adjuvant, increasing the PMF of drug-resistant bacteria by modulating both the transmembrane chemical gradient and the electrical potential across the membrane. This increased drug uptake and led to *Salmonella* cell death.

## Data availability statement

The original contributions presented in this study are included in the article/Supplementary material, further inquiries can be directed to the corresponding author.

## Ethics statement

The animal study was approved by the Animal Ethics Committee of South China Agricultural University. The study was conducted in accordance with the local legislation and institutional requirements.

## Author contributions

CZ carried out the main experiments and data analysis and wrote the manuscript. YZ and JK participated in the *in vitro* validation tests. HY and JL participated in the isolation and identification of clinical drug-resistant bacteria. BF conceived and designed the experiments. All authors contributed to the article and approved the submitted version.
