# Supplementary material for: Alkaline arginine promotes the gentamicin-mediated killing of drug-resistant Salmonella by increasing NADH concentration and proton motive force
Source: Front Microbiol. 2023 Sep 19;14:1237825. doi: 10.3389/fmicb.2023.1237825 (PMC10546041; doi:10.3389/fmicb.2023.1237825)
Supplement: Supplementary file 2 [file Data_Sheet_1.PDF]

Supplementary Material

Article Title

First Author*, Co-Author, Co-Author

*** Correspondence:** Corresponding Author: email@uni.edu

1 Supplementary Figures

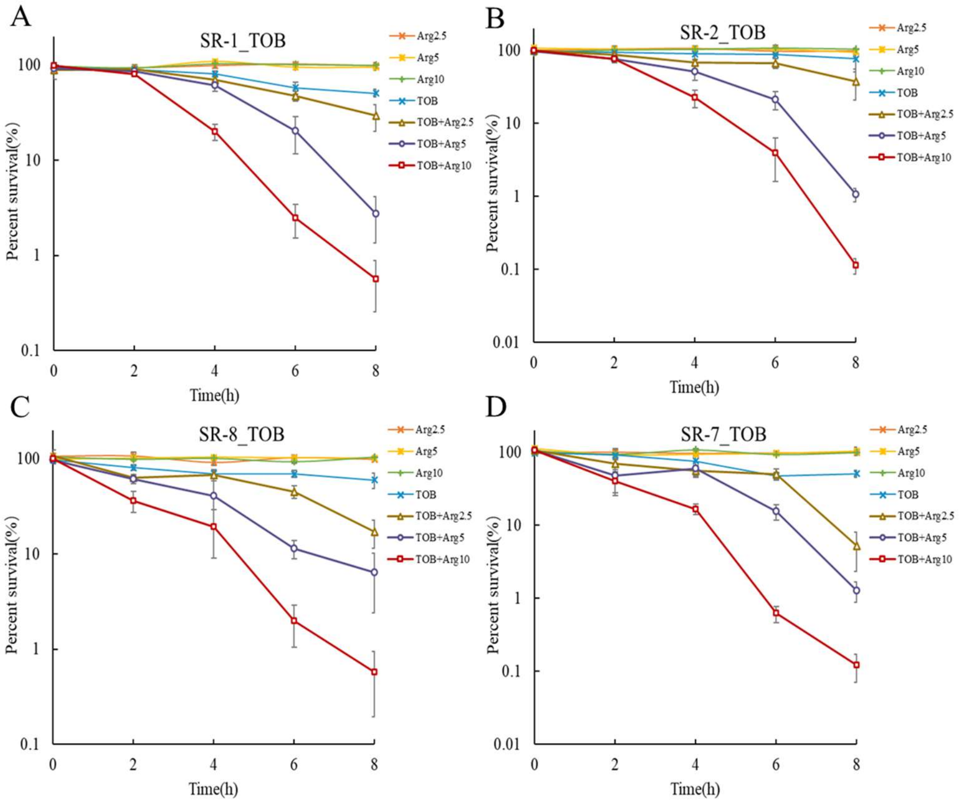

Supplementary Figure 1. Percent survival of clinical isolates SR-1 (A), SR-2 (B), SR-7 (C), and SR-8(D) in the presence of tobramycin (1MIC) by arginine dose.

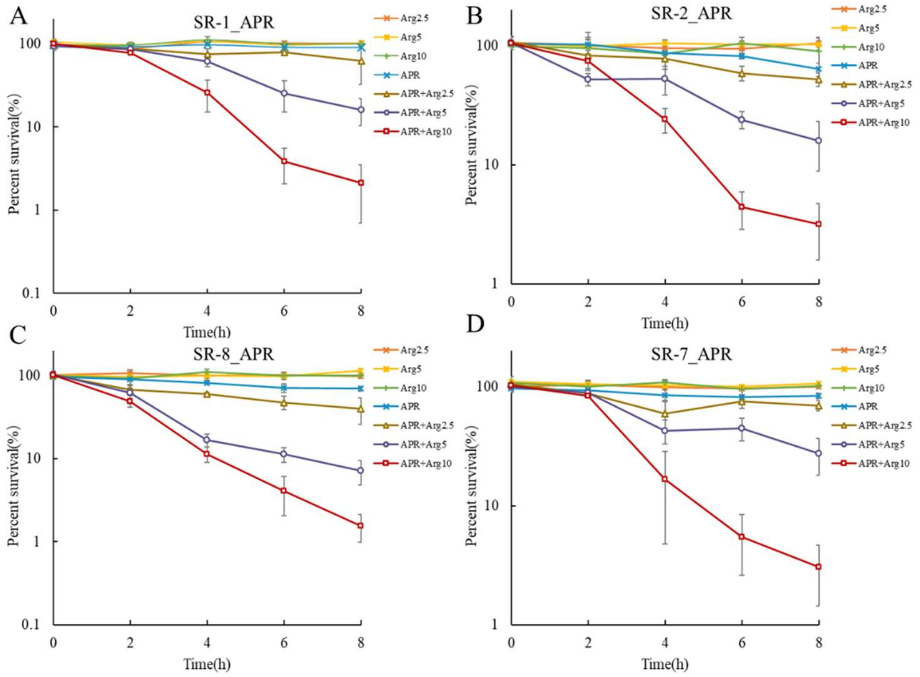

Supplementary Figure 2. Percent survival of clinical isolates SR-1 (A), SR-2 (B), SR-7 (C), and SR-8(D) in the presence of apramycin (1MIC) by arginine dose.

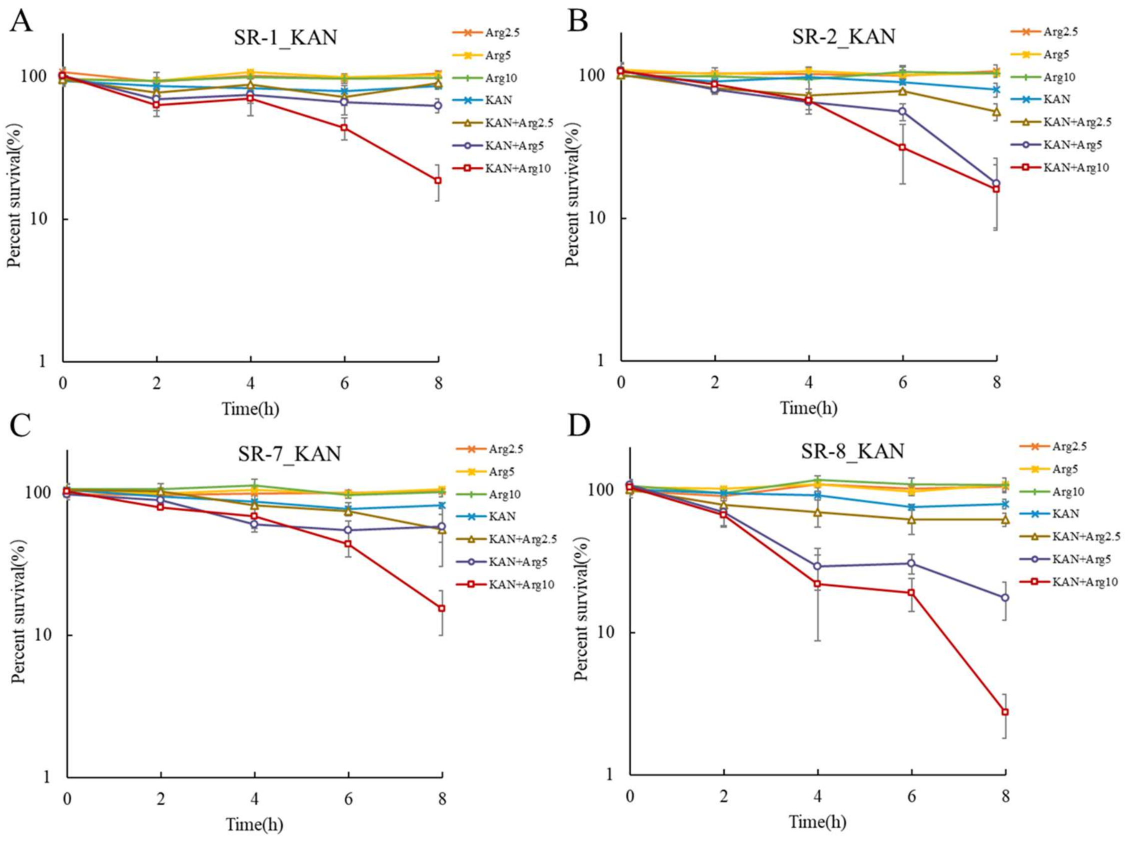

Supplementary Figure 3. Percent survival of clinical isolates SR-1 (A), SR-2 (B), SR-7 (C), and SR-8(D) in the presence of kanamycin (1MIC) by arginine dose.

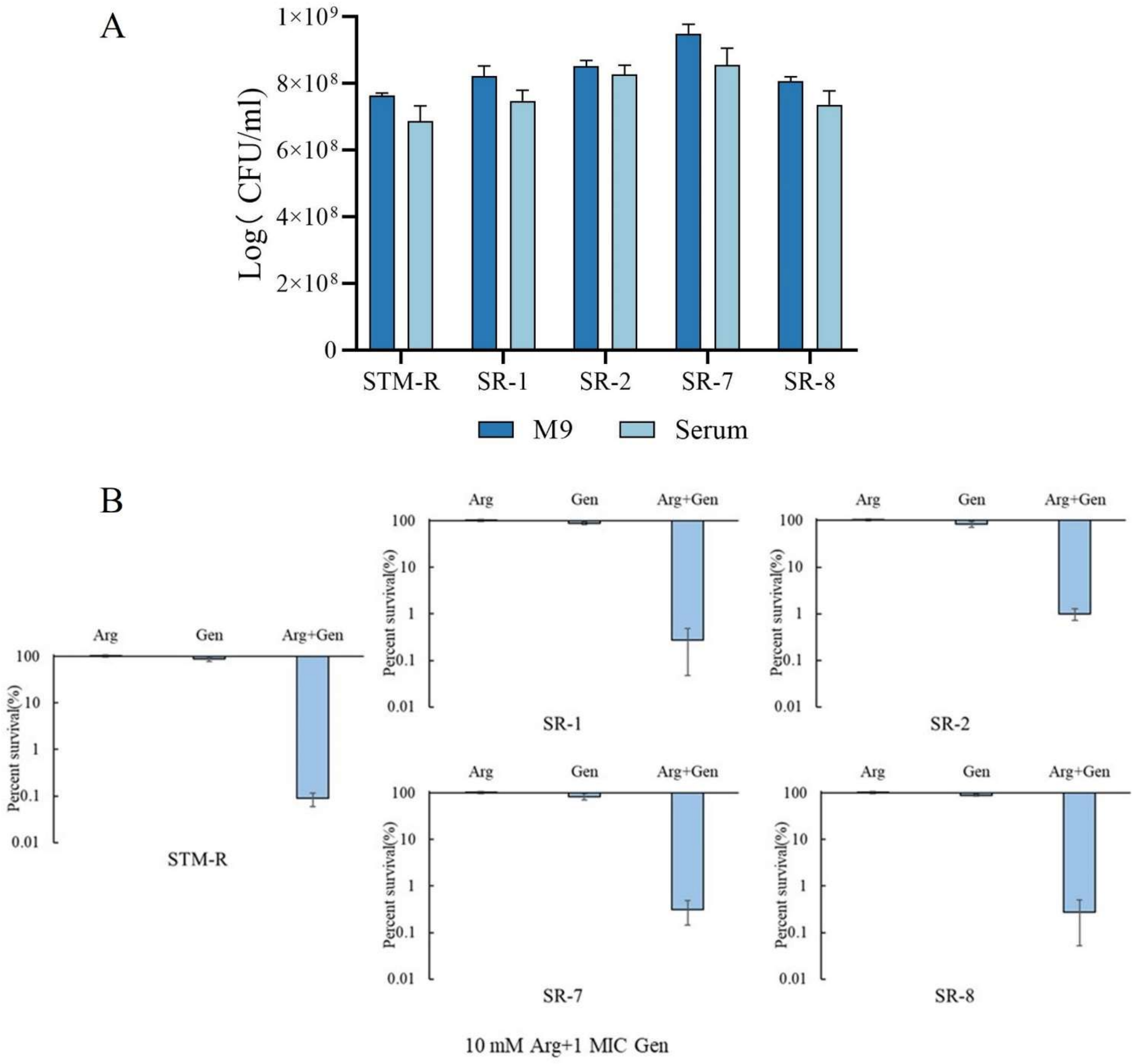

Supplementary Figure 4. The killing capacity of mouse serum alone (A). Percent survival of *Salmonella* in the presence of Gentamicin (1MIC) in mouse serum by arginine (10 mM) (B).

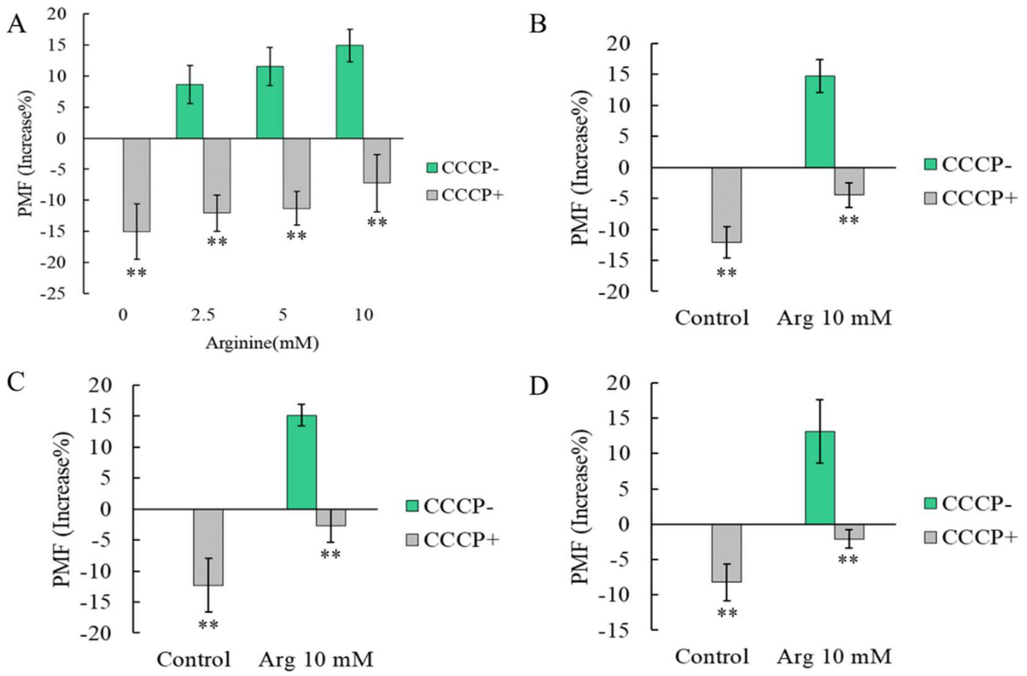

Supplementary Figure 5. Variation of PMF in SR-7 (A), SR-1 (B), SR-2 (C), and SR-8(D)by arginine dose with or without CCCP.

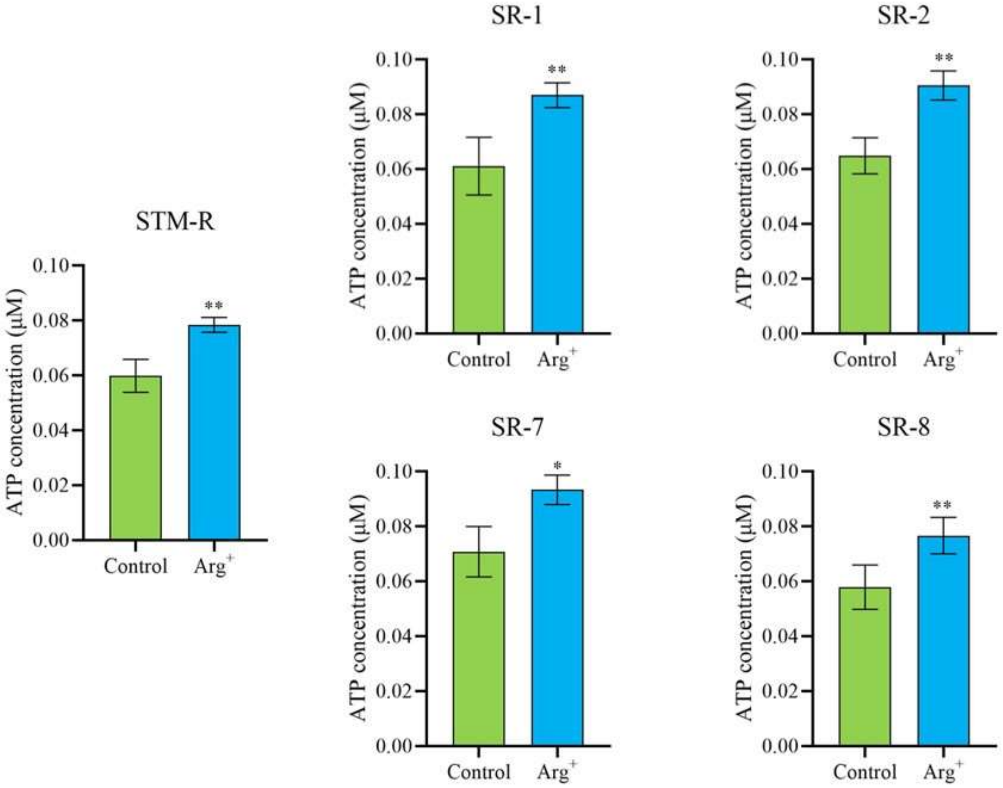

Supplementary Figure 6. Arginine increases the intracellular ATP concentration of STM-R and clinical drug-resistant *Salmonella* in the presence 10 mM arginine.

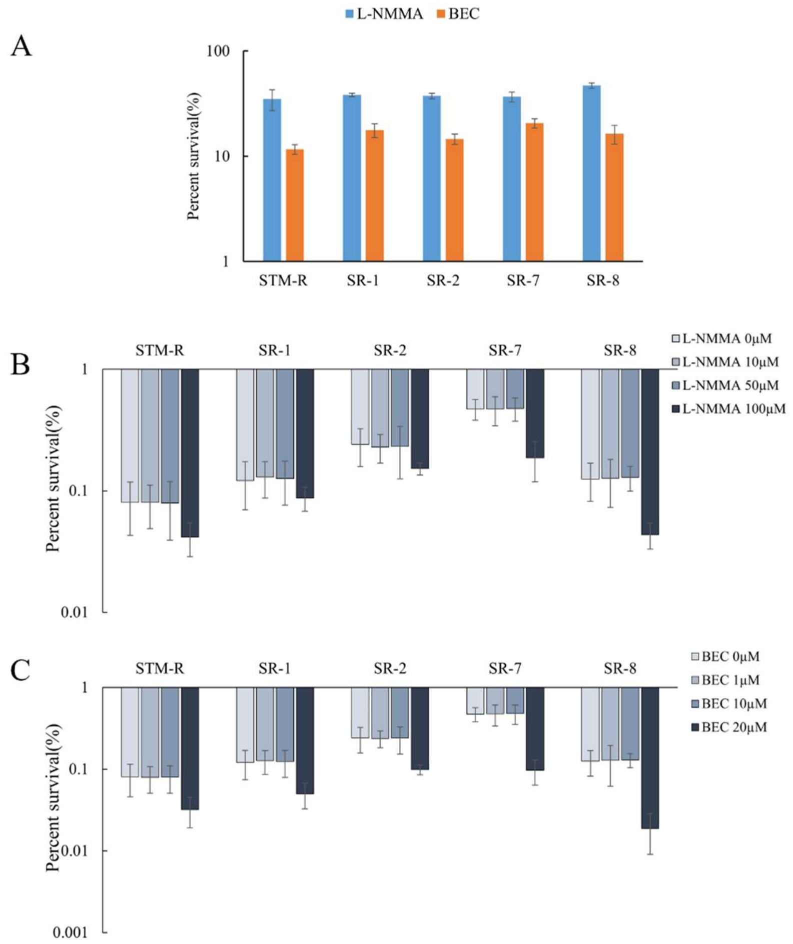

Supplementary Figure 7. Effect of arginine metabolic pathway inhibitor. Inhibitors of the arginine metabolic pathway. Minimum bactericidal concentration of arginase inhibitor (A) (L-NMMA 100mM, BEC20μM). Effects of arginase inhibitors and nitric oxide synthase inhibitors on synergistic effects. (B and C).
